# Strategies and Efforts towards the Total Synthesis of Palhinine Alkaloids

**DOI:** 10.3390/molecules25184211

**Published:** 2020-09-14

**Authors:** Yu-Yan Liang, Shi-Chao Lu, Ya-Ling Gong, Shu Xu

**Affiliations:** State Key Laboratory of Bioactive Substance and Function of Natural Medicines, and Beijing Key Laboratory of Active Substance Discovery and Druggability Evaluation, Institute of Materia Medica, Chinese Academy of Medical Sciences and Peking Union Medical College, 2A NanWei Road, Xicheng District, Beijing 100050, China; liangyuyan@imm.ac.cn (Y.-Y.L.); lushichao@imm.ac.cn (S.-C.L.); ylgong@imm.ac.cn (Y.-L.G.)

**Keywords:** *lycopodium* alkaloids, palhinine alkaloids, total synthesis.

## Abstract

The palhinine family of *Lycopodium* alkaloids were first reported in 2010, which feature an intriguing isotwistane carbon cage and a nine-membered azonane ring. It is noteworthy that the tetracyclic 5/6/6/9 skeleton was unprecedented in *Lycopodium* alkaloids before their seminal discovery. Over the past decade, extensive synthetic efforts stemming from seven research groups have resulted in two racemic total syntheses to date. This review article takes the opportunity to survey these efforts and achievements so as to promote further research towards the asymmetric total synthesis of palhinine alkaloids.

## 1. Introduction

**Figure 1:**
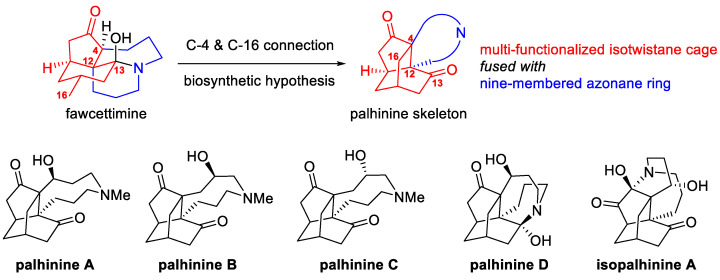
Five palhinine family *Lycopodium* alkaloids.

*Lycopodium* alkaloids [1] are a vast group of structurally related natural products isolated from the plants of the genus *Lycopodium*. They feature distinctive polyfused-bridged skeletons and impressive biological activities, including cytotoxicity, antimicrobial activity, anti-HIV-1 activity, enhanced mRNA expression for nerve growth factor, inhibition of pro-inflammatory factors, and inhibition of acetylcholinesterase (AChE). Particularly, the famous huperzine A, a potent reversible AChE inhibitor, is used as a drug for Alzheimer’s disease in China [2]. Since 2010, the palhinine family, a new subtype of *Lycopodium* alkaloids bearing more complex architectures, has been identified, which includes palhinines A–D and isopalhinine A (Figure 1). Their isolation from *Palhinhaea cernua* L. and *Lycopodium japonicum* Thunb. was achieved through the work of several prominent researchers, such as Wang and Long [3], Zhao [4,5], Yu [6], and others [7,8]. Structurally, palhinine alkaloids contain a multi-functionalized tricyclo[4.3.1.0^3,7^]decane ring (isotwistane cage) and a nine-membered azonane ring fused together through a bond between two neighboring all-carbon-substituted quaternary carbons (C-4 and C-12). Palhinines A–C [3,4] possess a 5/6/6/9 tetracyclic carbon ring system with different oxidation positions and configurations, while isopalhinine A [4] and palhinine D [6] have a more congested 5/6/6/6/7 aza-pentacyclic skeleton owing to the hemiaminalization of the carbonyl group and the secondary amine. The isotwistane skeleton was thought to originate biosynthetically from fawcettimine, a well-known *Lycopodium* alkaloid, through a new bridged connection between C-4 and C-16 [4]. Due to their scarcity in nature, palhinine alkaloids were reported to only be examined in limited bioassays, which showed no obvious activities. Therefore, due to this paucity, their synthesis has attracted the efforts of organic chemists who not only found their complex structure challenging, but worked to supply the community with a useful amount of the natural products for thorough pharmacological screening. During the ten years since the first isolation of the palhinine alkaloids was reported, seven research groups have reported their synthetic efforts towards these attractive natural products. Although synthesis of (±)-palhinines A and D and (±)-isopalhinine A has been accomplished through two distinct total synthesis strategies, no asymmetric total synthesis has been achieved thus far. This paper will survey these reported efforts and achievements in chronological order, with a special emphasis on strategies and key transformations. Through the contributions of these different research groups, we hope to provide a fuller picture of the evolution of the synthetic strategies employed toward this family of natural products.

## 2. Synthetic Efforts Reported during 2010–2015

**Figure 2:**
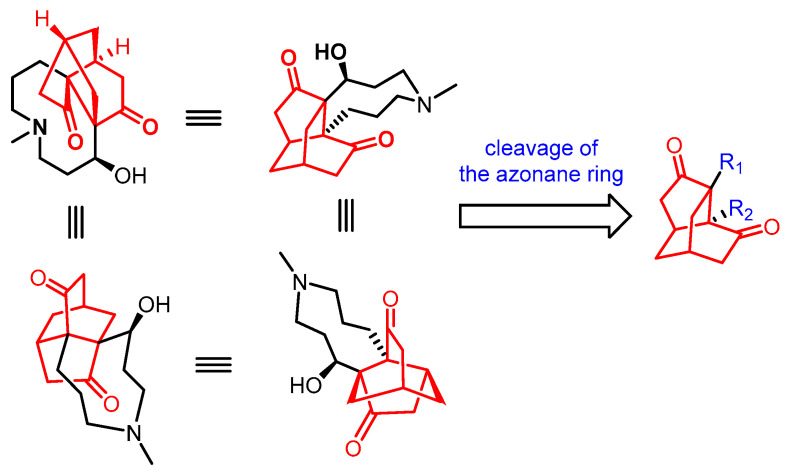
Retrosynthetic analysis of palhinine alkaloids (palhinine A as the example) in 2010–2015.

In the first five years since the first discovery of palhinine alkaloids, the synthetic strategies and efforts were reported from four groups. The research groups all planned to cyclize the nine-membered azonane ring in the late stages of their strategies (Figure 2) and primarily focused on the synthesis of the common isotwistane motif, which is obviously challenging and is a different feature from other synthetically well-studied fawcettimine-type *Lycopodium* alkaloids [9]. Though no total synthesis was accomplished during these studies, several elegant strategies were developed to produce the isotwistane motif.

### 2.1. Synthetic Studies from Xie and She’s Group (2012)

**Scheme 1:**
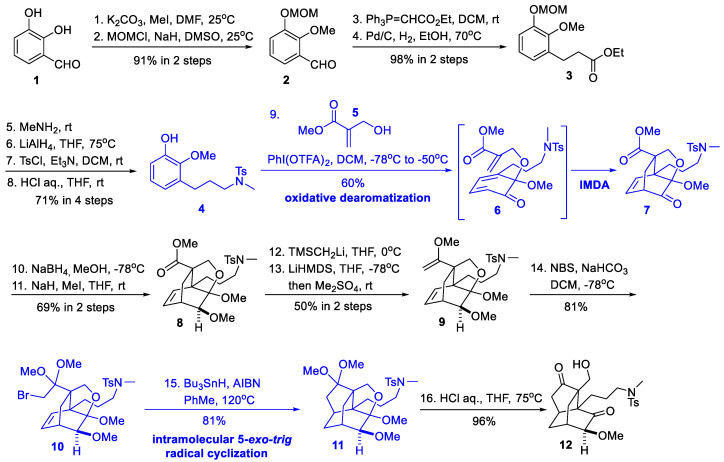
Synthesis of the isotwistane cage (Xie and She, 2012).

The first approach was disclosed by Xie and She and coworkers in 2012 [10]. Their key steps included early stage construction of the bicyclo[2.2.2]octane skeleton via a tandem oxidative dearomatization and intramolecular Diels–Alder (IMDA) reaction and a later intramolecular 5-exo-trig radical cyclization to form the tricyclic isotwistane cage (Scheme 1).

They first protected commercially available benzaldehyde derivative **1** according to a previously reported two-step procedure [11] to provide **2**, which was homologated to ester **3** through a Wittig reaction and subsequent hydrogenation. The key intermediate, phenol **4**, was furnished in 4 simple steps, employing an amidation, reduction, tosylation, and MOM deprotection protocol. Using PhI(OTFA)_2_, the oxidative dearomatization of **4** with hydroxymethylacrylate **5** generated ketal intermediate **6**, which in situ transformed to adduct **7** through a facile IMDA reaction. This key tandem reaction produced the desired bicyclo[2.2.2]octane skeleton and established the two neighboring stereogenic quaternary carbons. Sequential treatment of **7** with NaBH_4_ and MeI gave β-OMe-substituted **8**. Subsequently, three steps were required to homologate the methyl ester into bromoketal **10**. Finally, an intramolecular 5-exo-trig radical cyclization followed by two ketal hydrolysis delivered functionalized isotwistane cage **12**. The overall yield of **12** from **1** was 8.3% within 16 steps.

### 2.2. Synthetic Studies from Fan’s Group (2012)

**Scheme 2:**
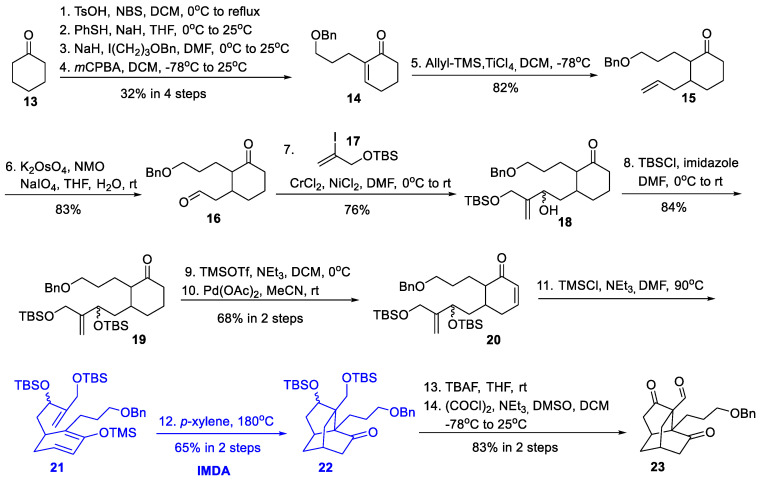
Synthesis of the isotwistane cage (Fan, 2012).

Later in 2012, Fan and coworkers reported their preliminary efforts towards the functionalized isotwistane cage, along with an IMDA strategy [12]. Differing from Xie and She’s procedure using two key steps, Fan’s group assembled the tricyclic isotwistane from a monocyclic precursor in a single step (Scheme 2).

Their synthesis began with the transformation of commercially available cyclohexanone (**13**) in 4 steps to enone **14**, in which an additional side chain was installed by Sakurai allylation, cleavage of olefin to aldehyde, and Nozaki-Hiyama-Kishi reaction with vinyl iodide **17**. A further three steps, including TBS protection, kinetic enolization–silylation, and Saegusa–Ito oxidation, provided cyclohexenone **20**. Thermodynamic enolization–silylation gave diene **21**, which underwent the key IMDA to isotwistane **22** in good yield. Subsequent deprotection and Swern oxidation provided **23** with the same functionalization pattern on the isotwistane cage as the natural target palhinine A. The overall yield of **23** from **13** was 5.1% within 14 steps.

### 2.3. Synthetic Studies from Maier’s Group (2013)

**Scheme 3:**
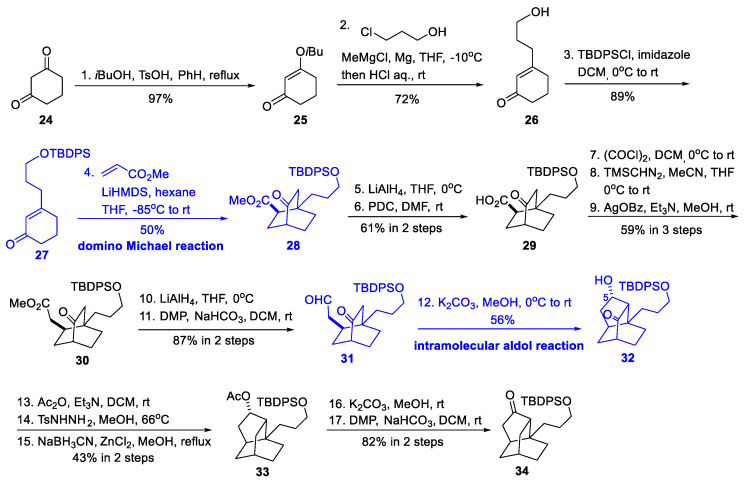
Synthesis of the isotwistane cage (Maier, 2013).

Maier and coworkers reported their studies on the synthesis of functionalized isotwistane cage **34** in 2013 [13]. Their synthesis was characterized by two key steps: a domino Michael reaction and an intramolecular aldol reaction (Scheme 3).

The synthesis was initiated by the formation of cyclohexenone **27** from dione **24** via a previously reported enol etherification [14], a Stork–Danheiser alkylation, and a TBDPS protection. The next key domino Michael reaction of **27** with methyl acrylate afforded a single diastereomer of **28**, not only constructing the core bicyclo[2.2.2]octane motif of the isotwistane cage, but also establishing the desired configuration of the ester side chain. Subsequent homologation of this side chain to aldehyde **31** was achieved via a seven-step procedure, including redox changes and an Arndt–Eistert protocol. An intramolecular aldol reaction cyclized the final five-membered ring of the isotwistane cage, affording a mixture of alcohol **32** and its epimer at C-5. Only the isomer **32** could be transformed to the final ketone **34** successfully via acetyl protection, Caglioti-modified Wolff–Kishner reduction, deacetylation, and Dess–Martin oxidation. The overall yield of **34** from **24** was 1.9% within 17 steps.

### 2.4. Synthetic Studies from Rychnovsky’s Group (2014)

**Scheme 4:**
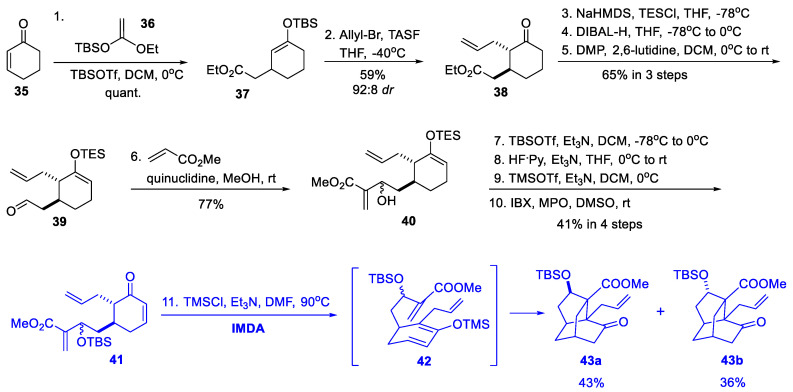
Synthesis of the isotwistane cage (Rychnovsky, 2014).

In 2014, Rychnovsky and coworkers independently reported an IMDA strategy similar to the one reported by Fan’s group to construct the isotwistane cage (Scheme 4) [15]. Initially, they rapidly installed two side chains to cyclohexanone (**35**) using a Mukaiyama−Michael allylation sequence. A further three steps were required to convert ester **38** into aldehyde **39**, which was subsequently reacted with methyl acrylate via a Morita–Baylis–Hillman reaction to provide **40** as a 1:1 mixture of epimers. The mixture was subsequently subjected to protecting group adjustment and oxidation to give enone **41**, which was also given as a mixture of epimers. Finally, unlike Fan’s two-step manipulation (**20** to **22**, Scheme 2), the thermodynamic enolization–silylation and the key IMDA reaction of **41** occurred efficiently at 90 °C in a single step and afforded the two corresponding epimers of isotwistane **43a** and **43b** in 43% and 36% yields, respectively. The combined yield of **43a** and **43b** from **35** was 8.8% within 11 steps.

## 3. Synthetic Efforts Reported during 2016–2020

The synthetic studies in 2010–2015 showed the efficiency of constructing the isotwistane skeleton of the palhinine alkaloids. Each group’s characteristics have enriched the methodologies used to achieve the assembly of the 5/6/6 ring system. According to their retrosynthetic analysis (Figure 2), the next task was to establish the nine-membered azonane ring using the protocols accumulated in the previous total synthesis of fawcettimine-type *Lycopodium* alkaloids. However, in 2016–2020, an unexpected great challenge was reported for the cyclization of the azonane ring embedded in the isotwistane cage of palhinine alkaloids. During the trial and error process, talent alternative ideas were generated and attempted from different groups, resulting in the accomplishment of two distinct total synthesis strategies.

### 3.1. Synthetic Studies and Total Synthesis from Fan’s Group (2016, 2017)

**Scheme 5:**
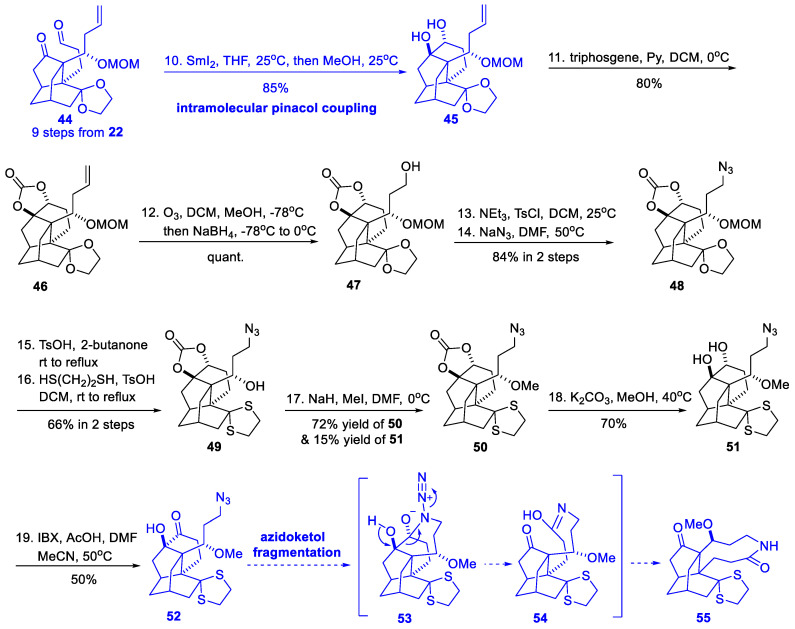
Attempt to synthesize a nine-membered azonane ring (Fan, 2016).

Based on their report in 2012 (Section 2.2), Fan’s group continued to study approaches to effect the azonane cyclization. Initially, several classical ring-closing tactics were employed, including *N*-alkylation, Mitsunobu reactions, and ring-closing olefin metathesis. However, none of those gave a nine-membered aza-cyclic product (see the supporting information in [16] for details). In 2016, the authors reported a preliminary attempt with intramolecular pinacol coupling and azidoketol fragmentation as key reactions (Scheme 5) [17].

The authors first prepared aldehyde **44** from their previously reported isotwistane **22** (Scheme 2) via a nine-step protocol [18]. The SmI_2_-mediated intramolecular pinacol coupling of **44** diastereoselectively provided diol **45** with a newly formed bridged bicycle. After protection of the vicinal diol as the carbonate, the terminal olefin was manipulated in three steps to azide **48**, which was one carbon shorter. Tactically, the glycol and MOM group of **48** was changed into acid-stable dithioketal and methyl ether in **50**. Further carbonate deprotection and IBX oxidation delivered the azidoketone **52**, which was proposed to transform into nine-membered azonane **55** via **53** and **54** by azidoketol fragmentation. Unfortunately, various attempts employing a range of Lewis acids or Brønsted acids gave no desired fragmentation.

**Scheme 6:**
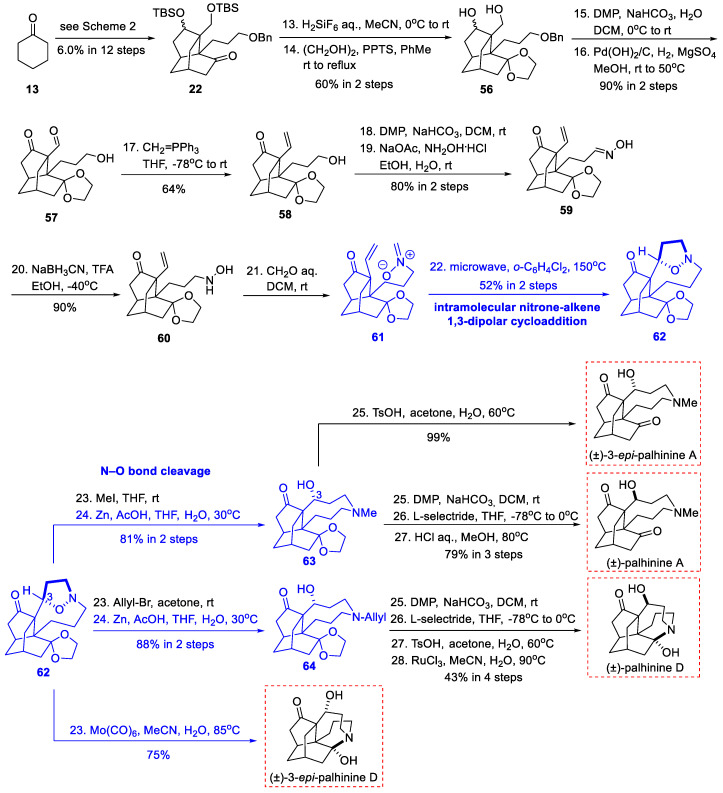
The first total syntheses of (±)-palhinine A and (±)-palhinine D (Fan, 2017).

Although the idea of pinacol coupling and azidoketol fragmentation failed, it was an attempt to use the auxiliary ring construction–deconstruction tactic to construct the nine-membered azonane ring. In 2017, with the same concept, Fan’s group reported a talent approach employing intramolecular nitrone−alkene 1,3-dipolar cycloaddition and subsequent N−O bond cleavage as key reactions, and accomplished the first total synthesis of (±)-palhinine A and (±)-palhinine D [16] (Scheme 6).

Once again using their previously reported isotwistane **22** (Scheme 2), they prepared aldehyde **57** in four steps through variation of the protecting group and oxidation state (Scheme 6). The nitrone–alkene **61** was then synthesized via a selective Wittig olefination and a four-step nitrone installation. Under microwave conditions, the key intramolecular nitrone−alkene 1,3-dipolar cycloaddition proceeded in a highly regio- and stereoselective manner providing the hexacyclic intermediate **62**, which contained the challenging nine-membered ring and an extra isoxazolidine auxiliary ring. The oxygen bridging linker was thought to alleviate the potential transannular strain in the nine-membered medium-sized ring. Subsequent *N*-methylation and N−O bond reductive cleavage dismantled the isoxazolidine auxiliary ring and provided **63**, which contains the full skeleton of palhinine alkaloids. Although the nitrone−alkene cyclization was highly selective, it gave the wrong configuration at the oxygen-substituted C-3 position compared with the natural target. This configuration can be converted into the natural one via DMP oxidation of **63** and a selective reduction using L-selectride. Finally, ketal deprotection accomplished the first total synthesis of (±)-palhinine A in 27 steps, achieving an overall yield of 0.49%. Similarly, palhinine D could be synthesized in 28 steps and an overall yield of 0.31% was achieved via *N*-allyl-substituted **64**. The 3-*epi*-palhinines A and D were also synthesized directly from intermediates **63** and **62**, respectively.

### 3.2. Synthetic Studies from She’s Group (2016)

**Scheme 7:**
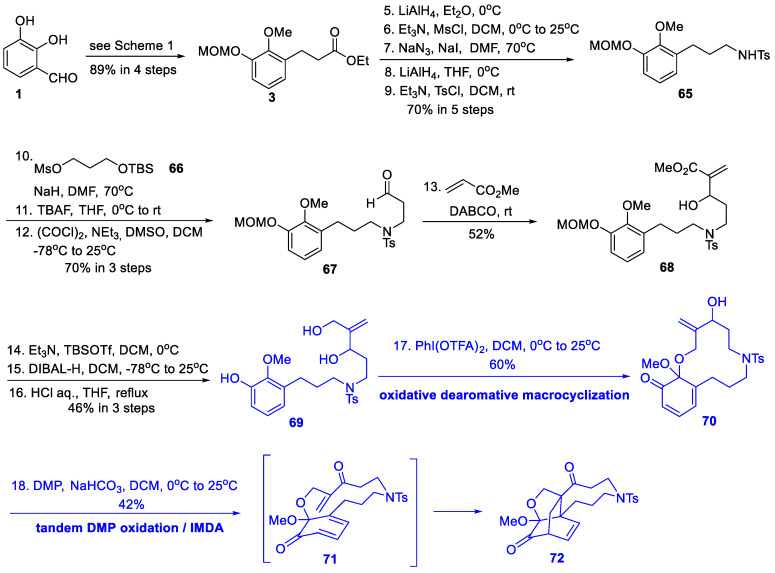
Synthesis of the nine-membered azonane ring with bicyclo[2.2.2]octane (She, 2016).

Following their successful synthesis in 2012 (Section 2.1), She and coworkers also made many attempts to install the azonane ring on the isotwistane cage, and also encountered great problems. Sandwiching between Fan’s reports in 2016 and 2017, She’s group disclosed their innovative and distinct approach in 2016 [19]. Different from Fan’s idea, She and coworkers proposed another talent strategy using ring constriction from a large macrocyclic ring system to overcome the inherent strain of the nine-membered medium-sized azonane ring (Scheme 7).

Ts-protected amine **65** was prepared in five steps from the previously reported intermediate **3** (Scheme 1). A subsequent *N*-alkylation employing **66**, TBS deprotection, and Swern oxidation gave aldehyde **67**, which was transformed into diol **69** in 4 steps, including a Morita–Baylis–Hillman reaction and other functional group manipulations. Subsequent oxidative dearomative macrocyclization afforded diene **70** with a 12-membered aza-ring. To narrow the energy level between diene and dienophile, **70** was oxidized by DMP to unsaturated ketone **71**, which in situ spontaneously afforded adduct **72** through an IMDA reaction. This key tandem reaction simultaneously installed two neighboring quaternary stereogenic carbons, one nine-membered azonane ring, and one bicyclo[2.2.2]octane, which is the core structure of the isotwistane cage. The overall yield of **72** from **1** was 2.7% within 18 steps.

### 3.3. Total Synthesis from Hsieh’s Group (2018)

**Scheme 8:**
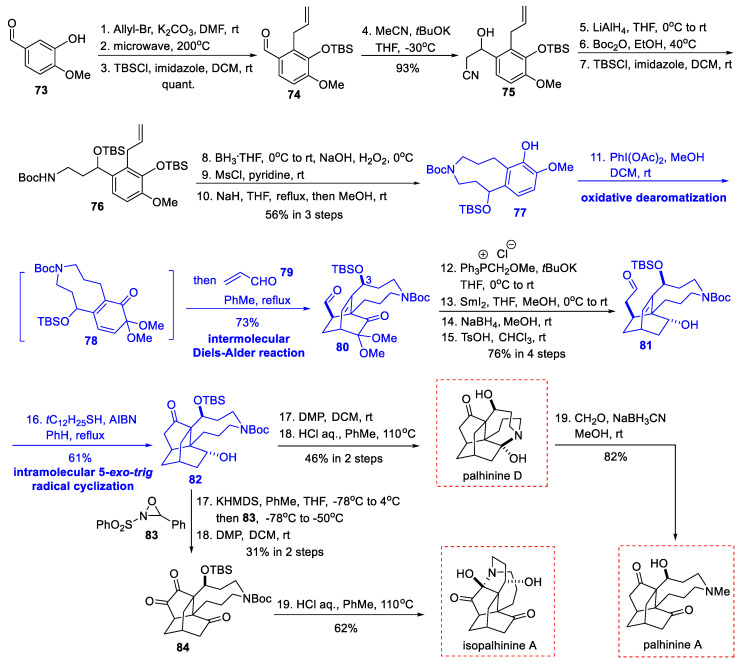
Total syntheses of (±)-palhinine A, (±)-palhinine D, and (±)-isopalhinine A (Hsieh, 2018).

In 2018, the first total synthesis of (±)-isopalhinine A together with the total synthesis of (±)-palhinine A and (±)-palhinine D was reported by Hsieh and coworkers [20]. To relieve the transannular strain, their elegant strategy installed the nine-membered azonane ring prior to the construction of the isotwistane cage (Scheme 8).

Initially, commercially available isovanillin (**73**) was used to produce aldehyde **74** via *O*-allylation, Claisen rearrangement, and TBS protection. The aza side chain was introduced via nucleophilic addition of MeCN, reduction to the amine, and subsequent protection. Further hydroboration–oxidation, mesylation, and intramolecular S_N_2 cyclization provided the nine-membered azonane **77**. Oxidative dearomatization and subsequent intermolecular Diels–Alder reaction with acrolein (**79**) in one pot provided the diastereoselective adduct **80** in 73% yield, together with 22% of its C-3 epimer. This key reaction not only achieved the azonane-embedded bicyclo[2.2.2]octane ring system, but also established the desired configuration of the aldehyde side chain. Subsequent homologation of this side chain and adjustment of the functional groups in 4 steps led to aldehyde **81**. Although cyclization of the last ring of isotwistane proved difficult at this stage, it was finally realized via an intramolecular thiol-mediated acyl radical cyclization to afford **82**. Next, Davis α-hydroxylation and DMP oxidation gave trione **84**. Further deprotection of TBS and Boc automatically gave the cyclized hemiaminal motif and accomplished the first total synthesis of (±)-isopalhinine A in 19 overall steps, achieving an overall yield of 2.1% from **73**. Further, (±)-palhinines D was also achieved from **82** by simple oxidation and deprotection. Further cleavage of the hemiaminal and *N*-methylation in one pot by reductive amination with formaldehyde succeeded in the biomimetic total synthesis of (±)-palhinine A (19 steps, 4.2% yield).

### 3.4. Synthetic Studies from Xu’s Group (2019)

**Scheme 9:**
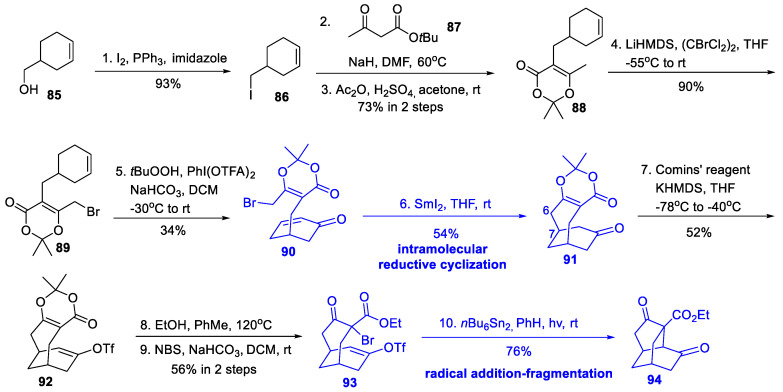
Synthesis of the isotwistane cage (Xu, 2019).

Despite the elegant strategies described above, an asymmetric total synthesis of palhinine alkaloids has not yet been reported. As all the studies described thus far have used Diels–Alder cycloaddition or the related domino Michael reaction to construct the bicyclo[2.2.2]octane of the isotwistane cage, we reasoned that this type of methodology might not be convenient for installing the initial chirality in palhinine synthesis. In 2019, our group proposed a non-Diels–Alder strategy and reported our preliminary results for construction of the isotwistane cage via SmI_2_-mediated reductive cyclization and light-initiated radical addition–fragmentation as key steps (Scheme 9) [21].

Starting with a commercially available alcohol **85**, we prepared cyclohexanone **90** in five steps by iodination, substitution with keto ester **87**, acetonide protection, γ-bromination, and allylic oxidation. Subsequently, the C-6 and C-7 connection was realized by a SmI_2_-mediated intramolecular reductive cyclization, providing a bicyclo[4.3.1]decane **91**. Next, regioselective enol-triflation, acetonide deprotection, and bromination resulted in bromide **93**. By UV irradiation in the presence of *n*Bu_6_Sn_2_, the C-4 and C-12 of **93** was bonded through a key radical addition–fragmentation reaction, affording isotwistane cage **94**. The overall yield of **94** from **85** was 2.5% within 10 steps.

### 3.5. Synthetic Studies from He’s Group (2020)

**Scheme 10:**
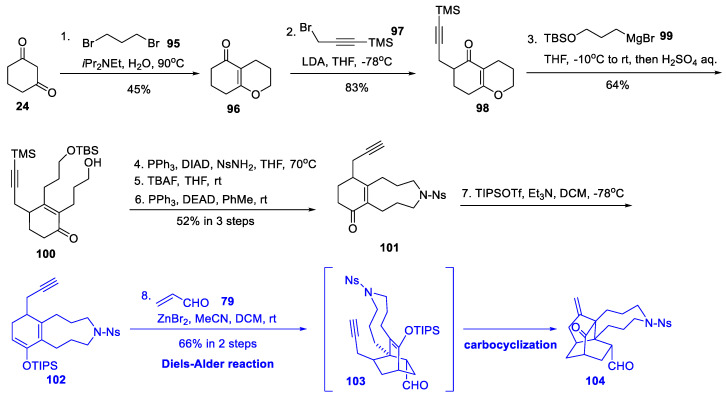
Synthesis of the azonane-ring-embedded isotwistane cage (He, 2020).

Very recently in 2020, He and coworkers reported the most concise route to construct the azonane-ring-embedded isotwistane cage [22]. The key reaction of their strategy was a highly efficient Lewis-acid-catalyzed Diels–Alder carbocyclization cascade (Scheme 10).

Their synthesis commenced with the transformation of dione **24** to cyclohexanone **98** via dialkylation and α-propargylation. Another side chain in **100** was then introduced by Stork–Danheiser alkylation and the nine-membered azonane cyclization in **101** was realized in three steps using the protocol of Fukuyama amine synthesis. After synthesis of the enol silane **102**, a ZnBr_2_-catalyzed Diels–Alder and carbocyclization cascade with acrolein (**79**) afforded **104** as a single diastereomer. This impressive key reaction not only generated four stereogenic centers, including two neighboring all-carbon substituted quaternary carbons, but also allowed the highly efficient construction of the isotwistane cage and the building up of all the rings for the common palhinine skeleton. The overall yield of this 8-step route is 8.2%.

## 4. Conclusions

**Figure 3:**
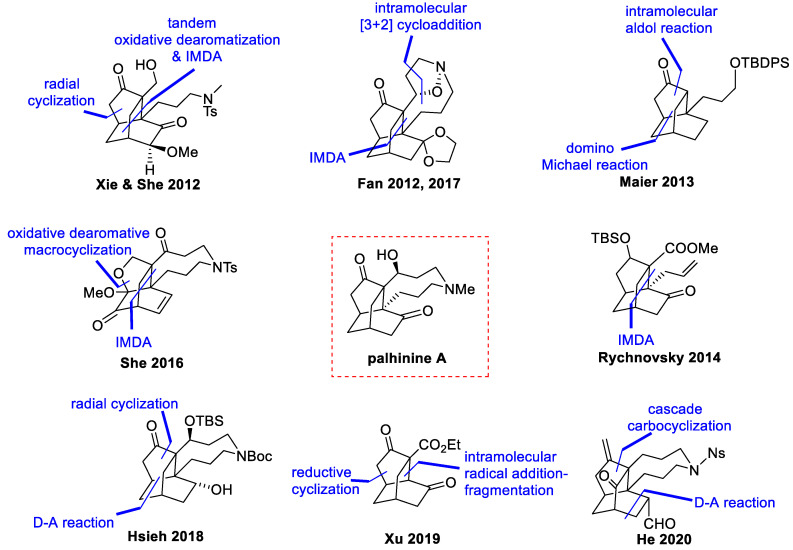
Overview of the reported key disconnections and intermediates towards the total synthesis of palhinine alkaloids.

This review summarizes all the reported studies toward the total synthesis of the palhinine family alkaloids since their isolation in 2010. During the decade since its discovery, diverse synthetic strategies and tactics have been developed to access this novel and challenging skeleton. A summary of the key synthetic cleavages, reactions, and advanced intermediates are shown in Figure 3. The research in the former half of the decade was focused on the construction of the synthetically demanding isotwistane cage, resulting in a series of diverse routes from four groups. Unexpectedly, subsequent studies revealed that installation of the nine-membered azonane ring onto the isotwistane cage was more challenging than initially thought. Classical methodologies employed in the total synthesis of *Lycopodium* alkaloids all proved to be unsuccessful. Therefore, in the latter half of the decade, the main theme was to overcome this problem, leading to the accomplishment of two different strategies and the achievement of the total syntheses of (±)-palhinine A, (±)-palhinine D, and (±)-isopalhinine A.

Despite the abovementioned success and significant progress, the asymmetric synthesis of these natural products has yet to be realized. All the reported studies are in racemic form, although some intermediates (**15** [23] in Scheme 2 and **85** [24] in Scheme 9) have been prepared in an enantioenriched form in other studies. Furthermore, the total synthesis of palhinines B and C, which contain the hydroxyl group at a different position, has not yet been achieved.

Overall, great efforts and achievements have been made over the last decade from several synthetic groups. There is still a need to provide a concise and asymmetric route applicable to this family of *Lycopodium* alkaloids. Rapid development and success in this field is anticipated in the near future.

## Abbreviation

Ac	acetyl
AIBN	2,2′-azobisisobutyronitrile
Bn	benzyl
Boc	*tert*-butoxycarbonyl
Bz	benzoyl
DABCO	1,4-diazabicyclo[2.2.2]octane
DCM	dichloromethane
DEAD	diethyl azodicarboxylate
DIAD	diisopropyl azodicarboxylate
DIBAL-H	diisobutylaluminium hydride
DMF	*N*,*N*-dimethylformamide
DMP	Dess–Martin periodinane
DMSO	dimethyl sulfoxide
IBX	*o*-iodoxybenzoic acid
IMDA	intramolecular Diels–Alder
KHMDS	potassium bis(trimethylsilyl)amide
LDA	lithium diisopropylamide
LiHMDS	lithium bis(trimethylsilyl)amide
*m*CPBA	*meta*-chloroperbenzoic acid
MOM	methoxymethyl
MPO	4-methyoxypyridine 1-oxide
Ms	methanesulfonyl
NaHMDS	sodium bis(trimethylsilyl)amide
NBS	*N*-bromosuccinimide
NMO	*N*-methylmorpholine oxide
Ns	2-nitrobenzenesulfonyl
PDC	pyridinium dichromate
Ph	phenyl
PPTS	pyridinium *p*-toluenesulfonate
Py	pyridine
quant.	quantitative yield
rt	room temperature
TASF	tris(diethylamino)sulfonium difluorotrimethylsilicate
TBAF	tetra-*n*-butylammonium fluoride
TBDPS	*tert*-butyldiphenylsilyl
TBS	*tert*-butyldimethylsilyl
TES	triethylsilyl
TFA	trifluoroacetyl
Tf	trifluoromethanesulfonyl
THF	tetrahydrofuran
TIPS	triisopropylsilyl
TMS	trimethylsilyl
Ts	*p*-toluenesulfonyl
